# Protocol for the development of guidance for stakeholder engagement in health and healthcare guideline development and implementation

**DOI:** 10.1186/s13643-020-1272-5

**Published:** 2020-02-01

**Authors:** Jennifer Petkovic, Alison Riddle, Elie A. Akl, Joanne Khabsa, Lyubov Lytvyn, Pearl Atwere, Pauline Campbell, Kalipso Chalkidou, Stephanie M. Chang, Sally Crowe, Leonila Dans, Fadi El Jardali, Davina Ghersi, Ian D. Graham, Sean Grant, Regina Greer-Smith, Jeanne-Marie Guise, Glen Hazlewood, Janet Jull, S. Vittal Katikireddi, Etienne V. Langlois, Anne Lyddiatt, Lara Maxwell, Richard Morley, Reem A. Mustafa, Francesco Nonino, Jordi Pardo Pardo, Alex Pollock, Kevin Pottie, John Riva, Holger Schünemann, Rosiane Simeon, Maureen Smith, Airton T. Stein, Anneliese Synnot, Janice Tufte, Howard White, Vivian Welch, Thomas W. Concannon, Peter Tugwell

**Affiliations:** 1 Bruyère Research Institute, Bruyère Continuing Care and University of Ottawa, 85 Primrose Ave East, Ottawa, Ontario Canada; 2 Department of Internal Medicine, American University of Beirut, Beirut, Lebanon; 3 Clinical Research Institute, American University of Beirut, Beirut, Lebanon; 4 McMaster University, Hamilton, Ontario Canada; 5 Bruyère Research Institute, 85 Primrose Ave East, Ottawa, Ontario Canada; 6 Nursing, Midwifery and Allied Health Professions (NMAHP) Research Unit, Glasgow Caledonian University, Glasgow, UK; 7 Faculty of Medicine, School of Public Health Imperial College, London, UK; 8 Agency for Healthcare Research and Quality, Rockville, MD USA; 9 Crowe Associates Ltd., Oxford, UK; 10 Department of Clinical Epidemiology, University of the Philippines-Manila, Taft Ave, 1000 Manila, Philippines; 11 American University of Beirut, Beirut, Lebanon; 12 National Health and Medical Research Council, Canberra, Australia; 13 School of Epidemiology and Public Health, University of Ottawa, Ottawa, Canada; 14 Ottawa Hospital Research Institute, Ottawa, Ontario Canada; 15 Department of Social & Behavioral Sciences, Richard M. Fairbanks School of Public Health, Indiana University, 1050 Wishard Blvd, RG 6046, Indianapolis, IN 46202 USA; 16 LLC/S.T.A.R. Initiative, Apple Valley, CA USA; 17 Departments of Obstetrics and Gynecology, Medical Informatics & Clinical Epidemiology, Emergency Medicine Oregon Health & Science, University School of Medicine and the OHSU-PSU School of Public Health, Portland, USA; 18 University of Calgary, Calgary, Canada; 19 School of Rehabilitation Therapy, Faculty of Health Sciences, Queen’s University, Kingston, Ontario Canada; 20 MRC/CSO Social & Public Health Sciences Unit, University of Glasgow, 200 Renfield Street, Glasgow, G2 3AX UK; 21 Alliance for Health Policy and Systems Research, World Health Organization, Geneva, Switzerland; 22 Cochrane Musculoskeletal Group, London, Ontario Canada; 23 University of Ottawa, Ottawa, Canada; 24 The Cochrane Collaboration, Cochrane Consumer Network, London, UK; 25 Department of Internal Medicine/Division of Nephrology and Hypertension, University of Kansas Medical Center, Kansas, USA; 26 Unit of Epidemiology and Statistics, IRCCS - Institute of Neurological Sciences of Bologna, Bologna, Italy; 27 Ottawa Hospital Research Institute, The Ottawa Hospital, Ottawa, Canada; 28 Nursing Midwifery and Allied Health Professions (NMAHP) Research Unit, Glasgow Caledonian University, Glasgow, UK; 29 Departments of Family Medicine and Epidemiology and Public Health, University of Ottawa, Ottawa, Canada; 30 Primary Care Research Group and Equity Methods Group, Bruyère Research Institute, Ottawa, Canada; 31 Department of Family Medicine, McMaster University, Hamilton, Ontario Canada; 32 Department of Health Research Methods, Evidence, and Impact, Cochrane Canada and McMaster GRADE Centre, Hamilton, Canada; 33 Cochrane Consumer Executive, Ottawa, Ontario Canada; 34 Programa de Pós-Graduação em Ciências da Saúde, Universidade Federal de Ciências da Saúde de Porto Alegre - UFCSPA, Rua Sarmento Leite 245, Porto Alegre, RS 90050-170 Brazil; 35 Centre for Health Communication and Participation, School of Psychology and Public Health, La Trobe University, Melbourne, Australia; 36 Cochrane Australia, School of Public Health and Preventive Medicine, Monash University, Melbourne, Australia; 37 PCORI, Seattle, USA; 38 Alfred Deakin University, Geelong, Australia; 39 Journal of Development Studies and Journal of Development Effectiveness, Geelong, Victoria Australia; 40 The Campbell Collaboration, Oslo, Norway; 41 The RAND Corporation, Boston, MA USA; 42 Tufts Clinical & Translational Science Institute, Tufts University School of Medicine, Boston, MA USA; 43 University of Ottawa, Department of Medicine, Faculty of Medicine, Ottawa, Canada; 44 Ottawa Hospital Research Institute, Clinical Epidemiology Program, Ottawa, Canada; 45 University of Ottawa, School of Epidemiology and Public Health, Faculty of Medicine, Ottawa, Canada

**Keywords:** Guidelines, Stakeholder engagement, Coproduction, Systematic reviews, Guidance, Equity, Integrated knowledge translation

## Abstract

**Background:**

Stakeholder engagement has become widely accepted as a necessary component of guideline development and implementation. While frameworks for developing guidelines express the need for those potentially affected by guideline recommendations to be involved in their development, there is a lack of consensus on how this should be done in practice. Further, there is a lack of guidance on how to equitably and meaningfully engage multiple stakeholders. We aim to develop guidance for the meaningful and equitable engagement of multiple stakeholders in guideline development and implementation.

**Methods:**

This will be a multi-stage project. The first stage is to conduct a series of four systematic reviews. These will (1) describe existing guidance and methods for stakeholder engagement in guideline development and implementation, (2) characterize barriers and facilitators to stakeholder engagement in guideline development and implementation, (3) explore the impact of stakeholder engagement on guideline development and implementation, and (4) identify issues related to conflicts of interest when engaging multiple stakeholders in guideline development and implementation.

**Discussion:**

We will collaborate with our multiple and diverse stakeholders to develop guidance for multi-stakeholder engagement in guideline development and implementation. We will use the results of the systematic reviews to develop a candidate list of draft guidance recommendations and will seek broad feedback on the draft guidance via an online survey of guideline developers and external stakeholders. An invited group of representatives from all stakeholder groups will discuss the results of the survey at a consensus meeting which will inform the development of the final guidance papers.

Our overall goal is to improve the development of guidelines through meaningful and equitable multi-stakeholder engagement, and subsequently to improve health outcomes and reduce inequities in health.

## Contribution to the literature

- Existing guidance on stakeholder engagement largely focuses on patient, consumer, caregiver, or family involvement in guideline development but does not provide guidance on multi-stakeholder engagement.
- The MuSE project will compile and provide evidence-based strategies for the equitable and meaningful engagement of multiple stakeholders during the guideline development and implementation process.
- The MuSE project will develop a stakeholder engagement plan for its own project stakeholders and evaluate its development and implementation.

## Background

Guidelines from various entities exist for many health conditions and health-related activities. Guidelines contain recommendations for health practice, public health, or health policy [1]. They are used by health care providers as well as policymakers, health system leaders, professional medical bodies, service organizations, funders, and regulatory authorities [2]. Recommendations in guidelines should be based on available research evidence [3]. Guideline development requires evaluating, summarizing, and making recommendations based on the available body of evidence regarding patient care, public health, and health systems. This requires weighing the benefits and risks that accompany all care and policy options before making recommendations [3]. Sonnad [4] noted that a lack of connection between guideline developers and those who use them often leads to controversy and uncertainty. Indeed, engaging guideline users during the guideline development process has been noted to improve guideline recommendation uptake [5, 6]. Schünemann and colleagues [7], for one, consider implementation in policy and practice as part of the guideline development process (steps 14 and 16). Throughout this work, we will use guideline development to encompass the guideline development and implementation processes.

In recent years, we have witnessed considerable shifts in how healthcare research is planned, delivered, shared, and evaluated. It is now increasingly expected that individuals or groups involved in or affected by health- and healthcare-related decisions, programs, or policies (termed “stakeholders”) should have a say in the planning, conduct, dissemination, uptake, and evaluation of healthcare research. In other words, stakeholders should be engaged in the entire process of guideline development. Several entities [8–11], including the World Health Organization (WHO) and the National Institute for Health and Care Excellence (NICE), recommend involving stakeholders in guideline development. As such, stakeholder engagement has become widely accepted in the production of trustworthy guidelines [12, 13]. In guideline development, stakeholder engagement is considered critical to ensuring priority guideline topics are identified and that comprehensive assessments of the evidence and other considerations are done [14–16].

Stakeholder involvement can help to ensure a guideline’s acceptability and feasibility to the end users. They can also ensure that equity and human rights issues are taken into consideration and support the adoption of its recommendations into policy and practice. This in turn may lead to improved adherence to any treatments and practices recommended [7, 17]. Stakeholder engagement in guideline development is part of a wider acceptance by the research community of the value of ensuring the participation of end users in the research and knowledge translation cycles [18, 19]. There is a moral imperative to engage end users in that people have a right to be involved in activities that may affect them. End user engagement may also improve the relevance, transparency, and usefulness of guidelines [20].

There are many stakeholder groups equally affected by recommendations in guidelines—e.g., patients, consumers, providers, general public, researchers, and policymakers. However, engagement with patients/public/community stakeholder groups dominates the literature, and guidance of the engagement with patient/public stakeholders is the most prominent [21–23]. In a review of guideline methodologies conducted by Armstrong and Bloom for example, patients/public stakeholders were consulted by 101 different guideline developers [21]. Many guideline groups that have sought to involve stakeholders have utilized limited numbers of participants or utilized slow and labor-intensive processes (e.g., time and resources needed to administer, collate, and respond to over 200 stakeholder views and comments) [12]. It is recognized that successful guideline development and implementation requires the engagement of multiple stakeholders [24] and “shared solutions” (input from patients, clinicians, and policymakers) improve health outcomes [24–26]. Patient/public stakeholders may potentially feel intimidated to contribute if they are only one voice among many. Keeping patient and public stakeholder voices separate from other stakeholder groups potentially shortchanges the input and influence that this group may offer. Equitable engagement of multiple stakeholder groups can help to ensure that guidelines contribute to reducing health disparities [27, 28]. However, there is a lack of consensus on how to identify and recruit relevant stakeholders, how they should be engaged, what their roles and responsibilities should be, how to evaluate the impact of their engagement in guideline development, and how to best collect and manage conflicts of interest as part of the engagement and guideline development process.

Schünemann et al. identified 18 steps in the guideline development process, based on a review of 35 guideline manuals published between 2003 and 2013 [7]. Several manuals mentioned the importance of including stakeholders, but few provided details on what stakeholder engagement should entail. In a review of 56 guidance documents for guideline development, 72% mentioned incorporating patients and their views in the process. However, the review did not provide sufficient detail on how to do this for each step of the guideline development process [29]. Armstrong et al. developed a framework for continuous patient engagement in clinical practice guideline development that outlines options for patient engagement in the steps in which they are most commonly involved [16]. While providing guidance on when to involve patients, the framework does not provide guidance on how to identify patients to participate and does not discuss other stakeholders.

Effective stakeholder engagement, ideally, facilitates the equitable contribution of relevant stakeholder groups in the guideline development and implementation process. This requires that guideline developers establish processes that prevent more financially powerful, highly vocal, or intellectually conflicted stakeholder groups from dominating the guideline development process. The Grading of Recommendations, Assessment, Development and Evaluation (GRADE) equity guidelines, for one, recommend the inclusion of underrepresented stakeholder groups in the guideline development and implementation process [30]. Stakeholders will have various levels of time, resources, and skills available to dedicate to the process, and ensuring that these differences do not result in certain stakeholder having more influence over the final guideline recommendations is important. A challenging aspect of engaging stakeholders is ensuring that interests are declared and that conflicts of interest are appropriately managed. Despite the importance of conflict of interest for guideline development, there is high variability in the process of disclosure and management of such conflicts across different organizations [27]. As a consequence, there are many inconsistencies in how stakeholder engagement is considered across guideline development groups. There should be opportunities for a variety of opinions to be heard, but it is vital that the recommendations made are objective and not unduly influenced by vested interests. Strategies such as active outreach activities, giving adequate time for comment on guideline recommendations and using processes with follow-up prompts that ensure all stakeholder comments are systematically addressed may assist in reducing potential inequities and increase guideline development transparency [12].

### Study aim

The study aim is to develop guidance for guideline developers that supports the equitable and meaningful engagement of multiple stakeholders throughout the guideline development and implementation process. Guideline development will be used to encompass development, implementation, and evaluation processes. The objectives, in terms of participants, interventions, and comparators, are stakeholder/stakeholder groups engaged in guideline development, engagement in the guideline development process, and no comparator. Outcomes are outlined below for each phase of the project.

### Key definitions

We define below the terms guideline, stakeholder, stakeholder engagement, levels of engagement, and under-represented groups used for this project.

#### Guideline

Guidelines are “systematically developed evidence-based statements which assist providers, recipients, and other stakeholders informed decisions about appropriate health interventions” [31].

#### Stakeholder

A stakeholder is any “individual or group who is responsible for or affected by health- and healthcare-related decisions that can be informed by research evidence” [32]. Further, we acknowledge that some stakeholders may use guidelines to inform decision-making (CIHR terms these stakeholders, knowledge users), while others may have an interest in the recommendations for other reasons [32–34]. We define eight stakeholder groups for this project, namely (1) persons and the public (e.g., patients, their caregivers, families, and patient and consumer advocacy organizations), (2) providers (individuals/organizations that provide care, e.g., nurses, physicians, pharmacists, mental health counselors, community-based workers), (3) payers (pays for or reimburses for health-related interventions, e.g., insurers, individuals with deductibles, others responsible for reimbursement for health-related interventions), (4) purchasers (e.g., employers, self-insured, governments, and other entities responsible for underwriting the cost of care), (5) policymakers (policymaking entities such as governments and professional associations), (6) product makers (e.g., drug/device manufacturers), (7) principal investigators (e.g., researchers), and (8) the press (e.g., publishers, news media) [32, 33].

#### Engagement

“Engagement” refers to the approach to gather input or contribution from stakeholders “toward the development of a guideline, completion of any stages of a guideline, or dissemination, uptake or evaluation of a guideline and its recommendations” [35]. Engagement is considered multi-directional, resulting in “informed decision-making about the selection, conduct, and use of the research” [32]. Depending on the context, engagement may also be termed collaboration, involvement, or partnership [36]. Herein, we will use the term “stakeholder engagement.”

#### Levels of engagement (Table 1)

The extent to which stakeholders are engaged in the guideline development process can vary. We identify four levels of engagement (see Additional file 1), adapted from previous work [37–39]: (1) *Communication*—stakeholders receive information but have no role in contributing; (2) *Consultation*—stakeholders provide their views, thoughts, feedback, opinions, or experiences but without a commitment to act on them; (3) *Collaboration—*stakeholders are engaged to influence the production of the guidelines (e.g., commenting, advising, ranking, voting, prioritizing, and reaching consensus) but without direct control over decisions; and (4) *Coproduction—*stakeholders are equal members of the guideline development team and participate in all steps of the guideline development process. Members benefit from each other’s knowledge, skills, and perspectives and build relationships in an open, trusting, and transparent atmosphere that encourages learning from each other. With ongoing collaboration and engagement, all members have an equal opportunity to influence each aspect of the guideline development process [25, 35, 37, 40–42].

**Table 1:** Levels of engagement [37–39]

Level	Description	
Communication (level 1)	Stakeholders receive information. Stakeholders may be present but have no role in contributing.	e.g., “here’s what we are doing”
Consultation (level 2)	Stakeholders provide their views, thoughts, feedback, opinions, or experiences but without a commitment to act on them.	e.g., “What do you think about what we are doing?”
Collaboration (level 3)	Stakeholders are engaged to influence the production of guidelines (e.g., commenting, advising, ranking, voting, prioritizing, reaching consensus). Stakeholders provide information which directly influences the guideline process, but without direct control over decisions.	e.g., “Please get involved in what we are doing”
Coproduction (level 4)	Stakeholders are equal members of the guideline development team and participate in all steps of the guideline development process. Stakeholders work together in various roles throughout the guideline development process. Stakeholders make collaborative decisions to shape the guideline recommendations	e.g., “Let’s do it together”

#### Under-represented groups

Under-represented groups refer to those individuals or groups who may experience health inequities for reasons such as a lack of inclusion in research, health policy, or guideline development; barriers to access of health services; or because of other socially stratifying factors, such as their place of residence, race/ethnicity/culture/language, occupation, gender/sex, subject matter knowledge, religion, education, socioeconomic status, social capital, age, or other individual characteristics [27, 28].

## Study design and methods

We adapted our methods from the guidance for developing research reporting guidelines by Moher et al. [43] and our internal Terms of Reference for GRADE project groups document. Moher and colleagues recommend identifying the need for guidance, reviewing the literature, identifying participants, conducting a Delphi survey to gather opinions and set priorities, and holding a face-to-face consensus meeting. We have made a slight modification by adding key informant interviews to gather opinions on what should be included in the preliminary guidance. The conception and design of the study is the product of a global consortium for Multi-Stakeholder Engagement, entitled MuSE.

### The MuSE consortium

The MuSE consortium was established in 2015 and includes over 80 researchers and stakeholders in various countries including: Australia, Brazil, Canada, Germany, Italy, Lebanon, the Netherlands, the Philippines, Switzerland, the UK, and the USA. The team includes researchers, policymakers, guideline developers, research funders, clinicians, patients and patient representatives, and policymakers from various organizations including the Agency for Healthcare Research and Quality (AHRQ), the Campbell Collaboration, Cochrane, GRADE Working Group, Health Canada, Patient-Centered Outcomes Research Institute (PCORI), Research and Development (RAND) Corporation, the World Health Organization (WHO), Joanna Briggs Institute (JBI), and multiple universities. All team members share an interest in developing methods and approaches for involving patients and other stakeholders in health outcomes research [44]. The MuSE consortium includes three working groups with each undertaking projects related to the development of methods for involving multiple stakeholders in health outcomes research (see Additional file 2).

### Stakeholder engagement in the MuSE project

We will develop a stakeholder engagement framework for this study that outlines how we will engage and evaluate our own stakeholder engagement processes throughout the project, drawing on principles of realist evaluation to explore what works, for whom, why, and in what context [45]. We have adopted a co-production approach, drawing on the National Institute for Health Research (NIHR) guidance which pays attention to sharing power, including the perspectives and skills of all involved, respecting and valuing the knowledge of all, reciprocity, and building and maintaining relationships [25, 37, 40–42, 46]. We will work with our stakeholder to agree on the best ways to operationalize those key elements of co-production. We have assembled a large, international project team of co-investigators, collaborators, and stakeholders representing the various stakeholder groups as part of the MuSE consortium. All members of the consortium will be invited to provide advice and collaborate throughout the planning of each project stage, interpreting results, and coproducing the final guidance paper. To ensure accurate and transparent reporting of our stakeholder engagement throughout this project, we will follow the Guidance for Reporting Involvement of Patients and the Public (GRIPP2) checklist [47]. We will document and report on the methods used to engage our stakeholders, the results of the stakeholder engagement, the extent to which stakeholders’ input influenced the guidance development process and outcomes, and the lessons learned from the experience [47].

### GIN McMaster Guideline Development Checklist

This study will use the 18 steps contained in Schünemann et al.’s Guidelines 2.0 checklist, known as the GIN McMaster Guideline Development Checklist, for guideline development as its organizing framework [7]. It provides guideline developers with a comprehensive checklist of items linked to relevant resources and tools to facilitate the guideline development process. The GIN McMaster Guideline Development Checklist has been used by guideline developers in various settings and was used to develop extensions for specific aspects of guideline development such as adaptation and rapid guideline development [48–52]. The 18 steps begin with the organization, budgeting, and planning of the development process, and continue with steps for priority setting, guideline group membership, question generation, all the way to developing recommendations, dissemination, implementation, and evaluation of the guidelines. Item six on the checklist relates to consumer and stakeholder involvement. As opposed to identifying stakeholder engagement as one step along the multi-step process of guideline development, the MuSE study will seek to document evidence-based guidance for stakeholder engagement for each step in the guideline development process (see Additional file 3).

### Equity and the PROGRESS-Plus framework

The Canadian Institutes for Health Research (CIHR) expects that all research applicants will integrate gender and sex into their research designs when appropriate [53]. The MuSE project recognizes gender as an important social determinant of health and contributor to health inequities. Gender refers to “the socially constructed characteristics of women and men—such as norms, roles, and relationships of and between groups of women and men. It varies from society to society and can be changed” [54]. Sex is “the different biological and physiological characteristics of males and females, such as reproductive organs, chromosomes, hormones, etc.” [54]. Gender considerations will be integrated throughout the MuSE project as part of the project’s adoption of the PROGRESS-Plus framework for equity. The PROGRESS-Plus framework identifies socially stratifying factors that can contribute to health inequities [55]. We define health inequities as difference in health status that is avoidable, unfair, and unjust [56]. PROGRESS refers to Place of residence, Race/ethnicity/culture/language, Occupation, Gender/sex, Religion, Education, Socioeconomic status, and Social capital. The Plus extends the original framework to include personal characteristics associated with discrimination (e.g., age, disability), features of relationships (e.g., children of smoking parents), and time-dependent relationships (e.g., release from incarceration) [55]. We will use the framework to guide the integration of health equity considerations throughout the project. Below, we describe the project stages and the integration of equity in each.

### Project stages

**Fig. 1:**
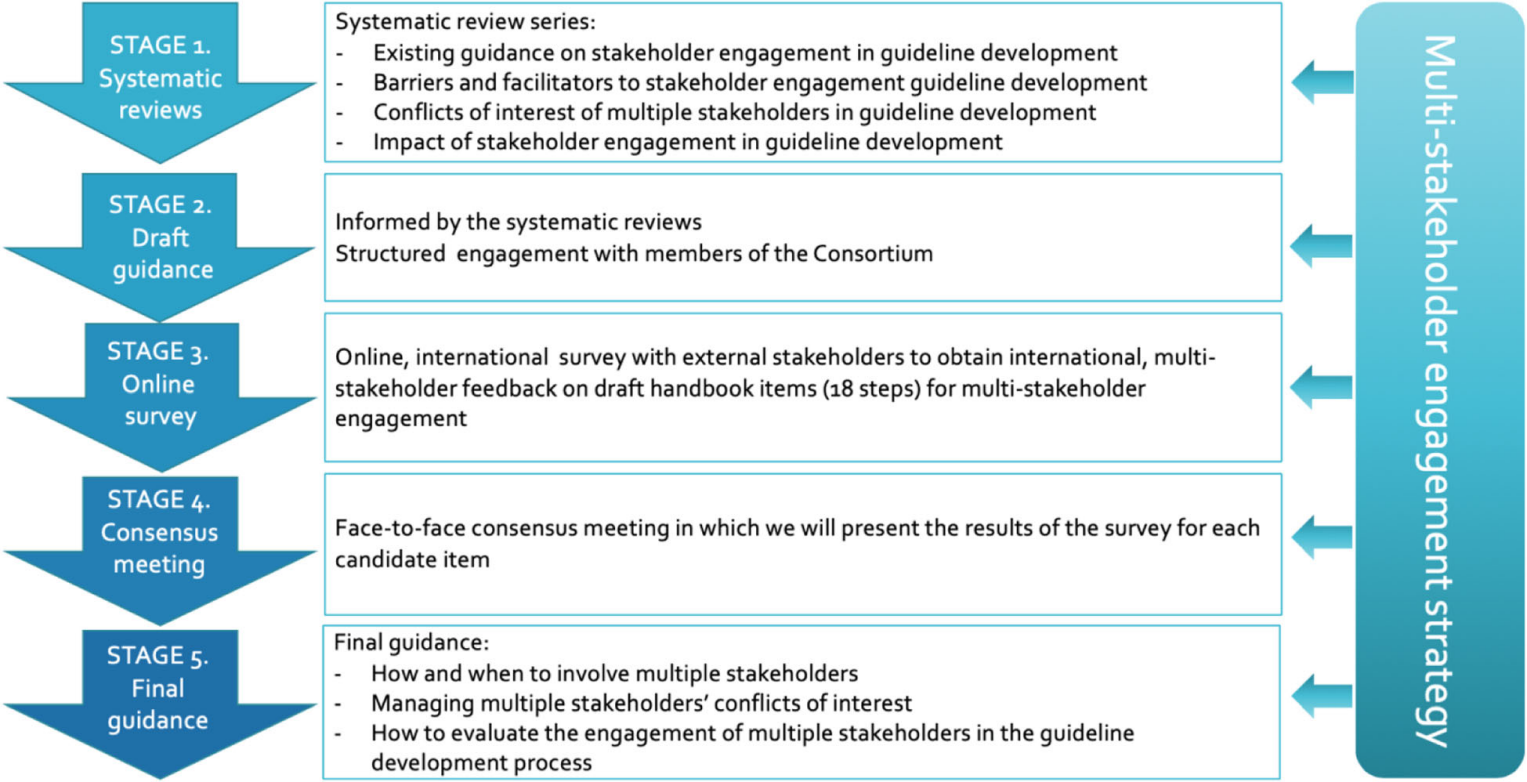
Project plan

This study includes various stages (Fig. 1). Briefly, they are (1) the conduct of four concurrent systematic reviews, (2) the development of draft guidance, (3) an online international survey of external stakeholders and experts, (4) a consensus meeting of project stakeholders, and (5) the finalization of the guidance paper. This protocol focuses on stage 1.

#### Stage 1: Systematic reviews of the literature

We will conduct four systematic reviews to identify and assess the available literature on stakeholder engagement in guideline development. This protocol will yield four distinct but related systematic reviews that will inform the overall protocol output. To improve efficiency, we will conduct an integrated literature search, screening, and data extraction process. Once included, articles will be tagged for their relevance to a particular review(s). Analyses and reports will be conducted separately for each review. Given the qualitative and multi-stage nature of this study, we believe the GRIPP2 checklist is more suitable for reporting the conduct of this project. The systematic review stage will be developed following the PRISMA-P checklist (Additional file 4)

Below, we briefly describe the objectives of each review and the methods common to all reviews.

Review 1: Existing guidance on stakeholder engagement in guideline development

The objective of this review is to synthesize existing guidance for stakeholder engagement at each of the 18 steps in the guideline development process. Eligible studies will describe the development of a framework or process for stakeholder engagement in guideline development. This includes frameworks or processes that address the implementation or evaluation of stakeholder engagement in guideline development. Outcomes to be synthesized are methods for (1) identifying stakeholders, (2) engaging stakeholders at different stages of the guideline development process, and (3) resolving differences in opinions/perspectives. In addition, frequency and level of engagement of stakeholders at each step of the guideline development process, level of engagement in each step of the guideline development process, and how stakeholders contributed at each step of the guideline development process will be synthesized.

Review 2: Barriers and facilitators to stakeholder engagement in guideline development

The expected result of this review is to summarize the barriers and facilitators to stakeholder engagement at each step in the guideline development process. Eligible studies will describe or assess the barriers and facilitators to stakeholder engagement in guideline development. A “barrier” is defined as any variable or condition that impedes stakeholder engagement in guideline development or implementation. A “facilitator” is defined as any variable or condition that promotes stakeholder engagement in guideline development or implementation.

Review 3: Conflicts of interest of multiple stakeholders in guideline development

The objective is to systematically review the literature on conflict of interest issues when engaging stakeholders in guideline development. Specifically, this review aims to answer the following questions: (1) What are the types of conflicts of interest that stakeholders engaged in the guideline development process have and how do they vary by stakeholder group? (2) Can the conflicts of interest of individuals or organizations selecting stakeholders to participate in the development of a guideline affect that selection process? (3) What are the potential effect(s) of conflicts of interest of stakeholders on the guideline development process? (4) What are the proposed and/or implemented approaches for managing the conflicts of interest of stakeholders engaged in guideline development, and what are their respective advantages and disadvantages?

Review 4: Impact of stakeholder engagement in guideline development

This review will assess the impact of stakeholder engagement in guideline development on (a) the guideline development process; (b) guideline relevance, trustworthiness, acceptability, and uptake; and (c) the stakeholders and panel members themselves. Eligible studies will identify or assess the impact of multi-stakeholder engagement through all the stages of guideline development.

##### Eligibility

Included studies will discuss stakeholder engagement in guideline development that assesses existing guidance and methods for stakeholder engagement at each stage of the guideline development and implementation process, barriers and facilitators to stakeholder engagement, conflicts of interest of stakeholders in the guideline development and implementation process, and/or impact of stakeholder engagement on the guideline development and implementation process.

##### Population

For our purposes, stakeholders in guideline development are as described above under the “Key definitions” section.

##### Intervention

Eligible studies will involve/engage stakeholders in some role(s) during the guideline development process. Engagement and guidelines are as described above under the “Key definitions” section.

##### Comparator

Studies that do not involve stakeholders in the guideline development process will not be eligible for inclusion in this project.

##### Study designs

All reviews will include quantitative, qualitative, and mixed-methods studies. As such, our methods will follow the Cochrane Handbook for Systematic Reviews of Interventions and the Handbook for Synthesizing Qualitative Research, as appropriate [57, 58]. We will include randomized trials, non-randomized studies (e.g., cohort studies, before and after studies, cross-sectional studies), qualitative studies, theoretical and ethical papers, process evaluation studies, policy analysis studies, case studies, and mixed-methods studies. We will exclude editorials, commentaries, proposals, and conference abstracts. Studies without a clear methods section will be excluded.

##### Search strategy

We will develop one comprehensive search strategy to identify relevant studies for the four reviews. We will search the following databases: MEDLINE (OVID), CINAHL (EBSCO), EMBASE (OVID), PsycInfo (OVID), AMED (OVID), and SCOPUS. We will not place limits on language, date, or study design. In addition, we will perform both backward and forward citation tracking to identify further eligible studies. A draft of the search strategy is provided (see Additional file 5).

To identify grey literature, we will search the websites of agencies who actively engage stakeholder groups such as the AHRQ, Canadian Institute of Health Research (CIHR) Strategy for Patient-Oriented Research (SPOR), INVOLVE, the National Institute for Health and Care Excellence (NICE), and the PCORI. We will also search the websites of guideline-producing agencies, such as the American Academy of Pediatrics, Australia’s National Health Medical Research Council (NHMRC), and the WHO. We will invite members of the team to suggest grey literature sources, and we plan to broaden the search by soliciting suggestions via social media, such as Twitter (https://twitter.com/CochraneEquity).

##### Study selection

Titles and abstracts of the studies identified by the search strategy and the full texts of those assessed as potentially relevant will be screened independently, in duplicate using Covidence software [38]. Disagreements on study selection will be resolved by discussion or with a third member of the research team when necessary. Eligible studies will be exported into an Excel spreadsheet and independently “tagged” for their relevance to each review. One study may be included in more than one review. For example, a study may describe a process for stakeholder engagement in guideline development as well as describe the barriers and facilitators to the stakeholder engagement.

##### Data extraction

The data extraction form will be pre-tested and will include (as applicable) factors related to the population, intervention, comparison, and outcomes. Extracted data common to all reviews include information on study characteristics (e.g., year of publication, authors, type of publication), stakeholders included, stakeholder roles, and stakeholder characteristics. The data will be extracted independently in duplicate by two reviewers and will be piloted on ten articles. Disagreements on extractions will be resolved by discussion and with a third member of the research team when necessary. Where necessary, the corresponding author of eligible studies will be contacted for additional information

We will use the 18 steps of the guideline development process as a framework for data extraction and will map the existing guidance, barriers and facilitators, conflicts of interest, and impacts discussed in our included studies to one or more of the steps of this framework [7]. Further, we will extract data that describe study populations and results or findings by PROGRESS-Plus factors.

##### Risk of bias

We will examine the methodological quality of the included studies as appropriate. We will use the risk of bias tools from the Cochrane Handbook for randomized trials (ROB2) [59], the Risk of Bias in Non-randomized Studies – of Interventions (ROBINS-I) tool [60], and the Critical Appraisal Skills Programme (CASP) qualitative appraisal research tool [61] for qualitative studies. Risk of bias will be assessed independently, in duplicate, by two authors, and any discrepancies will be resolved by consensus and consultation with a third author, when necessary.

##### Analyses

We will use a mixed-methods approach to summarize our findings. Qualitative and quantitative studies will be analyzed and synthesized separately, while the implications for practice, policy, and research that will form the discussion and conclusion sections of the reviews will draw on both the qualitative and quantitative syntheses. We will summarize findings across the 18-steps of the GIN-McMaster Checklist, the eight stakeholder groups, and the PROGRESS-Plus factors. We will report the systematic reviews following the Preferred Reporting Items for Systematic Reviews and Meta-Analysis (PRISMA) [62] and the Enhancing Transparency in Reporting the Synthesis of Qualitative Research (ENTREQ) [63] reporting guidelines, as appropriate. The GRADE or Confidence in the Evidence from Reviews of Qualitative Research (CERQual) methodology will be used to evaluate the quality of evidence for each review as appropriate.

#### Stage 2: Drafting the guidance

In consultation with the MuSE consortium, we will use the results of the four systematic reviews to develop a candidate list of recommendations to include in the final guidance paper. These draft recommendations will provide guidance mapped to the 18 steps of the guideline development process which will allow guideline developers to consider when and how to engage different stakeholders at each stage of the guideline development process. Equity considerations at each step of the guideline development process will be outlined.

Drafting and refining the guidance will involve structured engagement similar to that described by Jull and colleagues [64] with all MuSE consortium members. Because of our large and geographically diverse team, we will consult with members via email, teleconferences, and face-to-face meetings, as appropriate, to revise the guidance and ensure that all team members have equal opportunity to contribute and influence the research output. We will work with our MuSE consortium members to equitably include diverse voices in the drafting process, including visible minorities, women, members from low- and middle-income countries, and others who are traditionally omitted from guideline development processes.

## Discussion

Stakeholder engagement should be multidirectional, meaningful, effective, and enable equity for both the stakeholders and guideline developers throughout all steps in the decision-making process. This project is innovative in that we are committed to an inclusive, comprehensive, and equitable approach to ensure that the guidance we develop is representative and relevant for all stakeholders, including those who are involved in creating and implementing guidelines and those affected by the recommendations developed within those guidelines. Our stakeholder engagement strategy for the project will assist us in effectively engaging our own stakeholders and allow us to monitor our engagement processes in real-time so that course corrections can be made if there is evidence of non-meaningful engagement.

We are not including language or date restrictions on our search strategy. A potential limitation of these reviews is that we are including papers regardless of their methodological quality. This will allow us to collect and synthesize qualitative data that we may miss if standard methodological criteria are applied. We will discuss limitations further in the full review.

These reviews will contribute to the literature by identifying existing guidance, barriers and facilitators, potential impacts, and possible conflict of interest issues related to engagement of stakeholders from many stakeholder groups in guideline development and implementation.

The results of the four reviews will inform the development of draft guidance. Once drafted, we will gather opinions and priorities on the guidance items from a wide range of purposefully selected stakeholders external to the MuSE consortium, including representation from low- and middle-income countries through an anonymous, online survey. We will strive for equity in the identification of survey recipients by engaging with a diversity of respondents representing different physical capabilities, genders, geographies, socio-economic statuses, and ethnicities. We will then present the results of the survey for each candidate item and use structured discussions to reach consensus on the included items for the final guidance paper at a two-day face-to-face consensus meeting as recommended by Moher et al. [43]. Finally, based on the results of the previous stages, we will develop guidance that provides recommendations for stakeholder roles and modes of engagement at different steps of the guideline development process (including implementation and evaluation) and for managing conflicts of interest. We will use an iterative process of feedback to draft, refine, and finalize the guidance to be provided in each manuscript in consultation with the co-authors of each paper and the other members of the MuSE consortium. The final product will be included in GRADE Working Group Guidance. The GRADE Working Group has developed internationally recognized guidance for the development of clinical practice and public health guidelines [65].

The expected final guidance will contribute to improving the guideline development and implementation process by identifying strategies for the meaningful and equitable engagement of all relevant stakeholder groups at all stages. Through this project, we aim to contribute to the growing body of literature on stakeholder engagement for better quality guidance, increased uptake of guidance, more relevant health programs, policies and services, and more equitable health outcomes.

We continually welcome additional expressions of interest and suggestions for relevant literature and plan to evaluate our own stakeholder engagement throughout this work to ensure meaningful engagement.

## Supplementary information

**Additional file 1: Table S1.** Levels of engagement.

**Additional file 2.** The MuSE Consortium.

**Additional file 3.** PRISMA-P reporting checklist.

**Additional file 4.** Stakeholders across the 18 steps.

**Additional file 5.** Draft Search Strategy in Medline.

## Data Availability

Data sharing is not applicable to this article as no datasets were generated or analyzed during the current study.

## Abbreviations

AHRQ: Agency for Healthcare Research and Quality
CASP: Critical Appraisal Skills Programme
CERQual: Confidence in the Evidence from Reviews of Qualitative Research
CIHR: Canadian Institute of Health Research
ENTREQ: Enhancing Transparency in Reporting the Synthesis of Qualitative Research
GRADE: Grading of Recommendations, Assessment, Development and Evaluation
GRIPP: Guidance for Reporting Involvement of Patients and the Public
JBI: Joanna Briggs Institute
MuSE: Multi-stakeholder engagement
NICE: National Institute for Health and Care Excellence
NIHR: National Institute for Health Research
PCORI: Patient-Centered Outcomes Research Institute
PRISMA: Preferred Reporting Items for Systematic Reviews and Meta-Analysis
PROGRESS: Place of residence, Race/ethnicity/culture/language, Occupation, Gender/sex, Religion, Education, Socioeconomic status, and Social capital
ROB: Risk of bias
ROBINS-I: Risk of Bias in Non-randomized Studies – of Interventions
SPOR: Strategy for Patient-Oriented Research
WHO: World Health Organization
