# Supplementary material for: Protocol for the development of guidance for stakeholder engagement in health and healthcare guideline development and implementation
Source: Syst Rev. 2020 Feb 1;9:21. doi: 10.1186/s13643-020-1272-5 (PMC6995157; doi:10.1186/s13643-020-1272-5)
Supplement: Supplementary file 1 — Additional file 1: Table S1. Levels of engagement. [file 13643_2020_1272_MOESM1_ESM.docx]

**Table 1. Levels of engagement**(37–39)

| Level | Description |  |
| --- | --- | --- |
| Communication (level 1) | Stakeholders receive information. Stakeholders may be present but have no role in contributing. | e.g. “here’s what we are doing” |
| Consultation (level 2) | Stakeholders provide their views, thoughts, feedback, opinions or experiences but without a commitment to act on them. | e.g. “What do you think about what we are doing?” |
| Collaboration (level 3) | Stakeholders are engaged to influence the production of guidelines (e.g. commenting, advising, ranking, voting, prioritising, reaching consensus). Stakeholders provide information which directly influences the guideline process, but without direct control over decisions. | e.g. “Please get involved in what we are doing” |
| Coproduction (level 4) | Stakeholders are equal members of the guideline development team and participate in all steps of the guideline development process. Stakeholders work together in various roles throughout the guideline development process. Stakeholders make collaborative decisions to shape the guideline recommendations | e.g. “Let’s do it together” |
