# Supplementary figures and images for: Protocol for the development of guidance for stakeholder engagement in health and healthcare guideline development and implementation
Source: Syst Rev. 2020 Feb 1;9:21. doi: 10.1186/s13643-020-1272-5 (PMC6995157; doi:10.1186/s13643-020-1272-5)

## Additional file 1: The MuSE Consortium


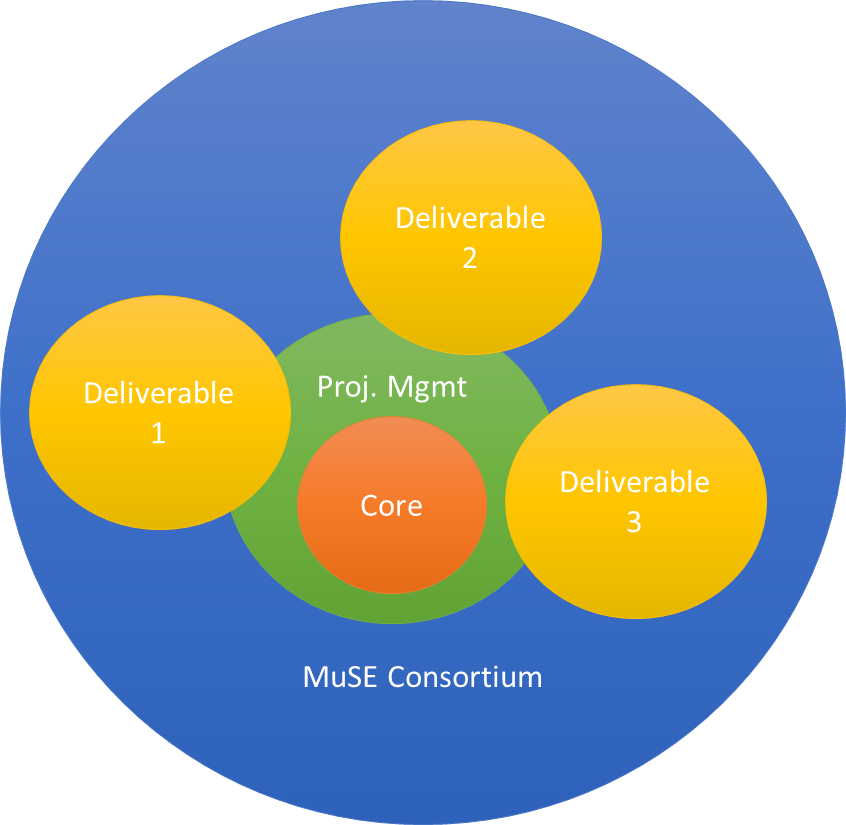

Supplement: Supplementary file 2 — Additional file 2. The MuSE Consortium. [file 13643_2020_1272_MOESM2_ESM.docx]
