# Supplementary material for: Protocol for the development of guidance for stakeholder engagement in health and healthcare guideline development and implementation
Source: Syst Rev. 2020 Feb 1;9:21. doi: 10.1186/s13643-020-1272-5 (PMC6995157; doi:10.1186/s13643-020-1272-5)
Supplement: Supplementary file 3 — Additional file 3. PRISMA-P reporting checklist. [file 13643_2020_1272_MOESM3_ESM.docx]

| MuSE 18 Topics in checklist for health and international guidance and guideline development  (adapted from Schunemann et al. 2014) | | Stakeholders | | | | | | | | | | | | | | | |
| --- | --- | --- | --- | --- | --- | --- | --- | --- | --- | --- | --- | --- | --- | --- | --- | --- | --- |
| Topic | Description | **Persons & the public** | **PROGRESS+** | **Provi-ders** | **PROGRESS+** | **Payers** | **PROGRESS+** | **Purcha-sers** | **PROGRESS+** | **Policy-makers** | **PROGRESS+** | **Product makers** | **PROGRESS+** | **Principal investig-ators** | **PROGRESS+** | **Press** | **PROGRESS+** |
| 1. Organization, budget, planning and training | Involves laying out a general but detailed plan describing what is feasible, how it will be achieved, and what resources are required to produce and use the guidance or guideline. The plan should refer to a specific period and be expressed in formal, measurable terms. |  |  |  |  |  |  |  |  |  |  |  |  |  |  |  |  |
| 2. Priority setting | Refers to the identification, balancing and ranking of priorities by stakeholders. Priority setting ensures that resources and attention are devoted to those general areas where recommendations will provide the greatest benefit to the population, a jurisdiction, or a country. A priority-setting approach needs to contribute to future plans while responding to existing, potentially difficult circumstances. |  |  |  |  |  |  |  |  |  |  |  |  |  |  |  |  |
| 3. Guidance and guideline group membership | Defines who is involved, in what capacity, and how the members are selected for the guidance or guideline development and at other steps of the guidance and guideline enterprise. |  |  |  |  |  |  |  |  |  |  |  |  |  |  |  |  |
| 4. Establishing guidance and guideline group processes | Defines the steps to be followed, how those involved will interact and how decisions will be made. |  |  |  |  |  |  |  |  |  |  |  |  |  |  |  |  |
| 5. Identifying target audience and topic selection | Involves describing the potential users or consumers of the guidance and guideline and defining the topics to be covered in the guidance or guideline. |  |  |  |  |  |  |  |  |  |  |  |  |  |  |  |  |
| 6. Consumer and stakeholder involvement | Describes how relevant people or groups who are not necessarily members of the panel but are affected by the guidance or guideline will be engaged. |  |  |  |  |  |  |  |  |  |  |  |  |  |  |  |  |
| 7. Conflict of interest considerations | Focuses on defining and managing the potential divergence between an individual’s interest and his or her professional obligations that could lead to questioning whether the actions or decisions are motivated by gain, such as financial, academic advancement, revenue streams, or community standing. Financial or intellectual or other relationships that may affect and individual’s or organization’s ability to approach a question with an open mind are included. |  |  |  |  |  |  |  |  |  |  |  |  |  |  |  |  |
| 8. Question generation | Focuses on defining key questions the recommendations should address using the PICO (population/problem, intervention, comparison, outcome) framework, including the detailed population, intervention and outcomes that will be relevant for decision-making. |  |  |  |  |  |  |  |  |  |  |  |  |  |  |  |  |
| 9. Considering importance of outcomes and interventions, values, preferences and utilities | Includes integrating, in the process of developing the guidance or guideline, how those affected by its recommendations assess the possible consequence. These include: knowledge, attitudes, expectations, moral and ethical values, and beliefs; goals for life and well-being; prior experience with the intervention; preferences for and importance of desirable and undesirable outcomes; perceived impact of the problem or interventions on quality of life, well-being or satisfaction, and interactions between the work of implementing the intervention, the intervention itself, and other contexts the person may be experiencing; preferences for alternative courses of action; and preferences relating to communication content and styles, information and involvement in decision-making. This can be related to what in the economic literature is considered *utilities.* An intervention itself can be considered a consequence of a recommendation and a level of importance or value is associated with that. |  |  |  |  |  |  |  |  |  |  |  |  |  |  |  |  |
| 10. Deciding what evidence to include and searching for evidence | Focuses on laying out inclusion and exclusion criteria based on types of evidence, study designs, characteristics of the populations, interventions and comparators, and deciding how the evidence will be identified and obtained. It also includes but is not limited to evidence about values and preferences, local data and resources. |  |  |  |  |  |  |  |  |  |  |  |  |  |  |  |  |
| 11. Summarizing evidence considering additional information | Focuses on presenting evidence in a synthetic format to facilitate the development and understanding of recommendations. It also involves identifying and considering additional information relevant to the question under consideration. |  |  |  |  |  |  |  |  |  |  |  |  |  |  |  |  |
| 12. Judging quality, strength, or certainty of a body of evidence | Includes assessing the confidence one can place in the obtained evidence by transparently evaluating the obtained research and other evidence applying structured approaches. This may include, but is not limited to, evidence about baseline risk or burden of the problem, importance of outcomes and interventions, values, preferences and utilities, resource use (cost), and estimates of effects. |  |  |  |  |  |  |  |  |  |  |  |  |  |  |  |  |
| 13. Developing recommendations and determining their strength | Developing recommendations involves use of a structured analytic framework and a transparent and systematic process to integrate the factors that influence a recommendation. Determining the strength of the recommendations refers to judgments about how confident a guidance and guideline panel is that the implementation of a recommendations exerts more desirable than undesirable consequences. |  |  |  |  |  |  |  |  |  |  |  |  |  |  |  |  |
| 14. Wording of recommendations and of considerations about implementation, feasibility, and equity | Refers to choosing syntax and formulations that facilitate understanding and implementation of the recommendations. Such wording is connected to considerations about implementation, feasibility, and equity, which refer to the guidance and guideline panel’s considerations about how the recommendation will be used and what impact it may have on the factors described. |  |  |  |  |  |  |  |  |  |  |  |  |  |  |  |  |
| 15. Reporting and peer review | Reporting refers to how guidance or guidelines will be made public. Peer review refers to how the guidance or guideline document will be reviewed before its publication and how it can be assessed, both internally and externally, by stakeholder who were not members of the guidance or guideline development group. |  |  |  |  |  |  |  |  |  |  |  |  |  |  |  |  |
| 16. Dissemination and implementation | Focuses on strategies to make relevant groups aware of the guidance or guidelines and to enhance their uptake. |  |  |  |  |  |  |  |  |  |  |  |  |  |  |  |  |
| 17. Evaluation and use | Refers to formal and informal strategies that allow judgments about: evaluation of the guidance or guidelines as a process and product, evaluation of the use or uptake, or both; and evaluation of impact and whether or not the guidance or guideline leads to improvement in outcomes or other consequences. |  |  |  |  |  |  |  |  |  |  |  |  |  |  |  |  |
| 18. Updating | Refers to how and when guidance or guidelines requires revision because of changes in the evidence or other factors that influence the recommendations. |  |  |  |  |  |  |  |  |  |  |  |  |  |  |  |  |
