# Supplementary material for: Protocol for the development of guidance for stakeholder engagement in health and healthcare guideline development and implementation
Source: Syst Rev. 2020 Feb 1;9:21. doi: 10.1186/s13643-020-1272-5 (PMC6995157; doi:10.1186/s13643-020-1272-5)
Supplement: Supplementary file 5 — Additional file 5. Draft Search Strategy in Medline. [file 13643_2020_1272_MOESM5_ESM.docx]

Database: Ovid MEDLINE(R) and Epub Ahead of Print, In-Process & Other Non-Indexed Citations and Daily <1946 to November 15, 2018>

Search Strategy:

--------------------------------------------------------------------------------

1 Stakeholder Participation/ (231)

2 patient participation/ (23106)

3 consumer participation/ (15994)

4 Community-Based Participatory Research/ (3625)

5 (coproduction or co-production).ti,ab,kf. (1462)

6 ((stakeholder* or advisor* or reference* or expert* or consultation* or steering) adj2 (group* or panel*)).ti,ab,kf. (34824)

7 ((stakeholder* or patient* or consumer* or public or caregiver* or care-giver* or communit* or citizen* or user* or service-user* or end-user* or clinician* or doctor* or physician* or nurse* or policymaker* or policy-maker* or funder* or indust* or pharmaceutical) adj2 (engag* or involv* or input or participat* or collaborat*)).ti,ab,kf. (112904)

8 or/1-7 (179634)

9 practice guidelines as topic/ (106788)

10 Guideline*.ti. (68614)

11 Guidance.ti,kf. (16959)

12 Clinical guideline*.ti,ab,kf. (11424)

13 Clinical Practice Guideline*.ti,ab,kf. (12561)

14 or/9-13 (174713)

15 8 and 14 (6569)

Database: Embase <1974 to 2018 October 18>

Search Strategy:

--------------------------------------------------------------------------------

1 Stakeholder Participation/

2 patient participation/

3 consumer participation/

4 Community-Based Participatory Research/

5 (coproduction or co-production).ti,ab,kf.

6 ((stakeholder* or advisor* or reference* or expert* or consultation or steering) adj2 (group* or panel)).ti,ab,kf.

7 ((stakeholder* or patient* or consumer* or public or caregiver* or communit* or citizen* or user* or service-user* or end-user* or clinician* or doctor* or physician* or nurse* or policymaker* or funder* or industry or pharmaceutical) adj2 (engag* or involv* or input or participat*)).ti,ab,kf.

8 or/1-7

9 practice guidelines as topic/

10 Guideline*.ti.

11 Guidance.ti,kf.

12 Clinical guideline*.ti,ab,kf.

13 Clinical Practice Guideline*.ti,ab,kf.

14 or/9-13

15 8 and 14

| **#** | **Query** | **Limiters/Expanders** | **Last Run Via** | **Results** |
| --- | --- | --- | --- | --- |
| S11 | S5 AND S10 | Search modes - Boolean/Phrase | Interface - EBSCOhost Research Databases  Search Screen - Advanced Search  Database - CINAHL Complete | 3,577 |
| S10 | S6 OR S7 OR S8 OR S9 | Search modes - Boolean/Phrase | Interface - EBSCOhost Research Databases  Search Screen - Advanced Search  Database - CINAHL Complete | 98,559 |
| S9 | (clinical OR practice) N1 guideline* | Search modes - Boolean/Phrase | Interface - EBSCOhost Research Databases  Search Screen - Advanced Search  Database - CINAHL Complete | 76,380 |
| S8 | TI Guidance OR SU Guidance | Search modes - Boolean/Phrase | Interface - EBSCOhost Research Databases  Search Screen - Advanced Search  Database - CINAHL Complete | 8,719 |
| S7 | TI Guideline* | Search modes - Boolean/Phrase | Interface - EBSCOhost Research Databases  Search Screen - Advanced Search  Database - CINAHL Complete | 36,516 |
| S6 | MH Practice Guidelines | Search modes - Boolean/Phrase | Interface - EBSCOhost Research Databases  Search Screen - Advanced Search  Database - CINAHL Complete | 65,423 |
| S5 | S1 OR S2 OR S3 OR S4 | Search modes - Boolean/Phrase | Interface - EBSCOhost Research Databases  Search Screen - Advanced Search  Database - CINAHL Complete | 92,009 |
| S4 | (stakeholder* or patient* or consumer* or public or caregiver* or care-giver* or communit* or citizen* or user* or service-user* or end-user* or clinician* or doctor* or physician* or nurse* or policymaker* or policy-maker* or funder* or indust* or pharmaceutical) N2 (engag* or involv* or input or participat* or collaborat*) | Search modes - Boolean/Phrase | Interface - EBSCOhost Research Databases  Search Screen - Advanced Search  Database - CINAHL Complete | 78,623 |
| S3 | (stakeholder* OR advisor* OR reference* OR expert* OR consultation* OR steering) N2 (group* OR panel*) | Search modes - Boolean/Phrase | Interface - EBSCOhost Research Databases  Search Screen - Advanced Search  Database - CINAHL Complete | 14,207 |
| S2 | coproduction OR co-production | Search modes - Boolean/Phrase | Interface - EBSCOhost Research Databases  Search Screen - Advanced Search  Database - CINAHL Complete | 355 |
| S1 | MH Consumer Participation | Search modes - Boolean/Phrase | Interface - EBSCOhost Research Databases  Search Screen - Advanced Search  Database - CINAHL Complete | 16,306 |

Database: PsycINFO <1806 to November Week 3 2018>

Search Strategy:

--------------------------------------------------------------------------------

1 Client Participation/ (1878)

2 Stakeholder/ and Involvement/ (30)

3 Stakeholder/ and Participation/ (24)

4 coproduction.ti,ab. (157)

5 co-production.ti,ab. (344)

6 (stakeholder* adj2 group*).mp. [mp=title, abstract, heading word, table of contents, key concepts, original title, tests & measures] (1555)

7 (stakeholder* adj2 panel).mp. [mp=title, abstract, heading word, table of contents, key concepts, original title, tests & measures] (12)

8 (advisor* adj2 group*).mp. [mp=title, abstract, heading word, table of contents, key concepts, original title, tests & measures] (403)

9 (advisor* adj2 panel).mp. [mp=title, abstract, heading word, table of contents, key concepts, original title, tests & measures] (197)

10 (reference* adj2 group*).mp. [mp=title, abstract, heading word, table of contents, key concepts, original title, tests & measures] (3913)

11 (reference* adj2 panel).mp. [mp=title, abstract, heading word, table of contents, key concepts, original title, tests & measures] (41)

12 (expert* adj2 group*).mp. [mp=title, abstract, heading word, table of contents, key concepts, original title, tests & measures] (1571)

13 (expert* adj2 panel).mp. [mp=title, abstract, heading word, table of contents, key concepts, original title, tests & measures] (2624)

14 (consultation adj2 group*).mp. [mp=title, abstract, heading word, table of contents, key concepts, original title, tests & measures] (413)

15 (consultation adj2 panel).mp. [mp=title, abstract, heading word, table of contents, key concepts, original title, tests & measures] (11)

16 (steering adj2 group*).mp. [mp=title, abstract, heading word, table of contents, key concepts, original title, tests & measures] (135)

17 (steering adj2 panel).mp. [mp=title, abstract, heading word, table of contents, key concepts, original title, tests & measures] (1)

18 (stakeholder* adj2 engag*).mp. [mp=title, abstract, heading word, table of contents, key concepts, original title, tests & measures] (778)

19 (stakeholder* adj2 involv*).mp. [mp=title, abstract, heading word, table of contents, key concepts, original title, tests & measures] (1113)

20 (stakeholder* adj2 input).mp. [mp=title, abstract, heading word, table of contents, key concepts, original title, tests & measures] (126)

21 (stakeholder* adj2 participat*).mp. [mp=title, abstract, heading word, table of contents, key concepts, original title, tests & measures] (315)

22 (patient* adj2 engag*).mp. [mp=title, abstract, heading word, table of contents, key concepts, original title, tests & measures] (2689)

23 (patient* adj2 involv*).mp. [mp=title, abstract, heading word, table of contents, key concepts, original title, tests & measures] (5046)

24 (patient* adj2 input).mp. [mp=title, abstract, heading word, table of contents, key concepts, original title, tests & measures] (191)

25 (patient* adj2 participat*).mp. [mp=title, abstract, heading word, table of contents, key concepts, original title, tests & measures] (4845)

26 (consumer* adj2 engag*).mp. [mp=title, abstract, heading word, table of contents, key concepts, original title, tests & measures] (667)

27 (consumer* adj2 involv*).mp. [mp=title, abstract, heading word, table of contents, key concepts, original title, tests & measures] (903)

28 (consumer* adj2 input).mp. [mp=title, abstract, heading word, table of contents, key concepts, original title, tests & measures] (98)

29 (consumer* adj2 participat*).mp. [mp=title, abstract, heading word, table of contents, key concepts, original title, tests & measures] (593)

30 (public adj2 engag*).mp. [mp=title, abstract, heading word, table of contents, key concepts, original title, tests & measures] (846)

31 (public adj2 involv*).mp. [mp=title, abstract, heading word, table of contents, key concepts, original title, tests & measures] (983)

32 (public adj2 input).mp. [mp=title, abstract, heading word, table of contents, key concepts, original title, tests & measures] (73)

33 (public adj2 participat*).mp. [mp=title, abstract, heading word, table of contents, key concepts, original title, tests & measures] (988)

34 (caregiver* adj2 engag*).mp. [mp=title, abstract, heading word, table of contents, key concepts, original title, tests & measures] (261)

35 (caregiver* adj2 involv*).mp. [mp=title, abstract, heading word, table of contents, key concepts, original title, tests & measures] (576)

36 (caregiver* adj2 input).mp. [mp=title, abstract, heading word, table of contents, key concepts, original title, tests & measures] (168)

37 (caregiver* adj2 participat*).mp. [mp=title, abstract, heading word, table of contents, key concepts, original title, tests & measures] (822)

38 (communit* adj2 engag*).mp. [mp=title, abstract, heading word, table of contents, key concepts, original title, tests & measures] (3038)

39 (communit* adj2 involv*).mp. [mp=title, abstract, heading word, table of contents, key concepts, original title, tests & measures] (7065)

40 (communit* adj2 input).mp. [mp=title, abstract, heading word, table of contents, key concepts, original title, tests & measures] (136)

41 (communit* adj2 participat*).mp. [mp=title, abstract, heading word, table of contents, key concepts, original title, tests & measures] (5551)

42 (citizen* adj2 engag*).mp. [mp=title, abstract, heading word, table of contents, key concepts, original title, tests & measures] (515)

43 (citizen* adj2 involv*).mp. [mp=title, abstract, heading word, table of contents, key concepts, original title, tests & measures] (301)

44 (citizen* adj2 input).mp. [mp=title, abstract, heading word, table of contents, key concepts, original title, tests & measures] (21)

45 (citizen* adj2 participat*).mp. [mp=title, abstract, heading word, table of contents, key concepts, original title, tests & measures] (879)

46 (user* adj2 engag*).mp. [mp=title, abstract, heading word, table of contents, key concepts, original title, tests & measures] (808)

47 (user* adj2 involv*).mp. [mp=title, abstract, heading word, table of contents, key concepts, original title, tests & measures] (1358)

48 (user* adj2 input).mp. [mp=title, abstract, heading word, table of contents, key concepts, original title, tests & measures] (198)

49 (user* adj2 participat*).mp. [mp=title, abstract, heading word, table of contents, key concepts, original title, tests & measures] (818)

50 (service-user* adj2 engag*).mp. [mp=title, abstract, heading word, table of contents, key concepts, original title, tests & measures] (115)

51 (service-user* adj2 involv*).mp. [mp=title, abstract, heading word, table of contents, key concepts, original title, tests & measures] (505)

52 (service-user* adj2 input).mp. [mp=title, abstract, heading word, table of contents, key concepts, original title, tests & measures] (18)

53 (service-user* adj2 participat*).mp. [mp=title, abstract, heading word, table of contents, key concepts, original title, tests & measures] (124)

54 (end-user* adj2 engag*).mp. [mp=title, abstract, heading word, table of contents, key concepts, original title, tests & measures] (19)

55 (end-user* adj2 involv*).mp. [mp=title, abstract, heading word, table of contents, key concepts, original title, tests & measures] (53)

56 (end-user* adj2 input).mp. [mp=title, abstract, heading word, table of contents, key concepts, original title, tests & measures] (11)

57 (end-user* adj2 participat*).mp. [mp=title, abstract, heading word, table of contents, key concepts, original title, tests & measures] (25)

58 (clinician* adj2 engag*).mp. [mp=title, abstract, heading word, table of contents, key concepts, original title, tests & measures] (336)

59 (clinician* adj2 involv*).mp. [mp=title, abstract, heading word, table of contents, key concepts, original title, tests & measures] (514)

60 (clinician* adj2 input).mp. [mp=title, abstract, heading word, table of contents, key concepts, original title, tests & measures] (53)

61 (clinician* adj2 participat*).mp. [mp=title, abstract, heading word, table of contents, key concepts, original title, tests & measures] (274)

62 (doctor* adj2 engag*).mp. [mp=title, abstract, heading word, table of contents, key concepts, original title, tests & measures] (92)

63 (doctor* adj2 involv*).mp. [mp=title, abstract, heading word, table of contents, key concepts, original title, tests & measures] (201)

64 (doctor* adj2 input).mp. [mp=title, abstract, heading word, table of contents, key concepts, original title, tests & measures] (5)

65 (doctor* adj2 participat*).mp. [mp=title, abstract, heading word, table of contents, key concepts, original title, tests & measures] (154)

66 (physician* adj2 engag*).mp. [mp=title, abstract, heading word, table of contents, key concepts, original title, tests & measures] (240)

67 (physician* adj2 involv*).mp. [mp=title, abstract, heading word, table of contents, key concepts, original title, tests & measures] (548)

68 (physician* adj2 input).mp. [mp=title, abstract, heading word, table of contents, key concepts, original title, tests & measures] (28)

69 (physician* adj2 participat*).mp. [mp=title, abstract, heading word, table of contents, key concepts, original title, tests & measures] (551)

70 (nurse* adj2 engag*).mp. [mp=title, abstract, heading word, table of contents, key concepts, original title, tests & measures] (421)

71 (nurse* adj2 involv*).mp. [mp=title, abstract, heading word, table of contents, key concepts, original title, tests & measures] (695)

72 (nurse* adj2 input).mp. [mp=title, abstract, heading word, table of contents, key concepts, original title, tests & measures] (20)

73 (nurse* adj2 participat*).mp. [mp=title, abstract, heading word, table of contents, key concepts, original title, tests & measures] (865)

74 (policymaker* adj2 engag*).mp. [mp=title, abstract, heading word, table of contents, key concepts, original title, tests & measures] (27)

75 (policymaker* adj2 involv*).mp. [mp=title, abstract, heading word, table of contents, key concepts, original title, tests & measures] (24)

76 (policymaker* adj2 input).mp. [mp=title, abstract, heading word, table of contents, key concepts, original title, tests & measures] (3)

77 (policymaker* adj2 participat*).mp. [mp=title, abstract, heading word, table of contents, key concepts, original title, tests & measures] (9)

78 (funder* adj2 engag*).mp. [mp=title, abstract, heading word, table of contents, key concepts, original title, tests & measures] (3)

79 (funder* adj2 involv*).mp. [mp=title, abstract, heading word, table of contents, key concepts, original title, tests & measures] (2)

80 (funder* adj2 input).mp. [mp=title, abstract, heading word, table of contents, key concepts, original title, tests & measures] (0)

81 (funder* adj2 participat*).mp. [mp=title, abstract, heading word, table of contents, key concepts, original title, tests & measures] (3)

82 (industry adj2 engag*).mp. [mp=title, abstract, heading word, table of contents, key concepts, original title, tests & measures] (66)

83 (industry adj2 involv*).mp. [mp=title, abstract, heading word, table of contents, key concepts, original title, tests & measures] (117)

84 (industry adj2 input).mp. [mp=title, abstract, heading word, table of contents, key concepts, original title, tests & measures] (9)

85 (industry adj2 participat*).mp. [mp=title, abstract, heading word, table of contents, key concepts, original title, tests & measures] (73)

86 (pharmaceutical adj2 engag*).mp. [mp=title, abstract, heading word, table of contents, key concepts, original title, tests & measures] (9)

87 (pharmaceutical adj2 involv*).mp. [mp=title, abstract, heading word, table of contents, key concepts, original title, tests & measures] (37)

88 (pharmaceutical adj2 input).mp. [mp=title, abstract, heading word, table of contents, key concepts, original title, tests & measures] (1)

89 (pharmaceutical adj2 participat*).mp. [mp=title, abstract, heading word, table of contents, key concepts, original title, tests & measures] (12)

90 1 or 2 or 3 or 4 or 5 or 6 or 7 or 8 or 9 or 10 or 11 or 12 or 13 or 14 or 15 or 16 or 17 or 18 or 19 or 20 or 21 or 22 or 23 or 24 or 25 or 26 or 27 or 28 or 29 or 30 or 31 or 32 or 33 or 34 or 35 or 36 or 37 or 38 or 39 or 40 or 41 or 42 or 43 or 44 or 45 or 46 or 47 or 48 or 49 or 50 or 51 or 52 or 53 or 54 or 55 or 56 or 57 or 58 or 59 or 60 or 61 or 62 or 63 or 64 or 65 or 66 or 67 or 68 or 69 or 70 or 71 or 72 or 73 or 74 or 75 or 76 or 77 or 78 or 79 or 80 or 81 or 82 or 83 or 84 or 85 or 86 or 87 or 88 or 89 (54340)

91 Treatment Guidelines/ (6250)

92 Guideline*.ti. (7140)

93 Guidance.ti. (6898)

94 guidance.id. (18812)

95 (clinical adj1 guideline*).mp. [mp=title, abstract, heading word, table of contents, key concepts, original title, tests & measures] (2171)

96 (practice adj1 guideline*).mp. [mp=title, abstract, heading word, table of contents, key concepts, original title, tests & measures] (3983)

97 91 or 92 or 93 or 94 or 95 or 96 (35305)

98 90 and 97 (715)

***************************

Scopus

November 26, 2018

( ( TITLE-ABS-KEY ( coproduction OR co-production ) ) OR ( TITLE-ABS-KEY ( ( stakeholder* OR advisor* OR reference* OR expert* OR consultation*  OR steering ) W/2 ( group* OR panel* ) ) ) OR ( TITLE-ABS-KEY ( ( stakeholder* OR patient* OR consumer* OR public OR caregiver* OR care-giver* OR communit* OR citizen* OR user* OR service-user* OR end-user* OR clinician* OR doctor* OR physician* OR nurse* OR policymaker* OR policy-maker* OR funder* OR industr* OR pharmaceutical ) W/2 ( engag* OR involv* OR input OR participat* OR collaborat* ) ) ) ) AND ( ( TITLE ( guideline* ) ) OR ( TITLE ( guidance ) ) OR ( KEY ( guidance ) ) OR ( TITLE-ABS-KEY ( "clinical guideline*" OR "clinical practice guideline*" ) ) )

**Sociological Abstracts:**

Searched for: (((MAINSUBJECT.EXACT ("Audience participation") OR MAINSUBJECT.EXACT("Participation") OR MAINSUBJECT.EXACT("Stakeholders") OR MAINSUBJECT.EXACT("Community") OR MAINSUBJECT.EXACT("Citizen participation") OR MAINSUBJECT.EXACT("Consumers")) OR noft((stakeholder* OR advisor* OR reference* OR expert* OR consultation* OR steering) NEAR/2 (group* OR panel*)) OR noft((stakeholder* OR patient* OR consumer* OR public OR caregiver* OR care-giver* OR communit* OR citizen* OR user* OR service-user* OR end-user* OR clinician* OR doctor* OR physician* OR nurse* OR policymaker* OR policy-maker* OR funder* OR indust* OR pharmaceutical) NEAR/2 (engag* OR involv* OR input OR participat* OR collaborat*))) AND (MAINSUBJECT.EXACT("Guidelines") OR ti(Guideline*) OR noft(Guidance*) OR noft(Clinical NEAR/3 guideline*))) AND stype.exact("Scholarly Journals")

Bottom of Form
